# 

**Published:** 1889-02

**Authors:** 


### BOOKS RECEIVED.

Essentials of Surgery. By Edward Martin, A.M., M.D.

Essentials of Anatomy. By Charles B. Nancrede, M.D.

Essentials of Medical Chemistry. By Lawrence Wolff, M.D.

Essentials of Obstetrics. By William Easterly Ashton, M.D.

The Waters of Plombières (Vosges). By Dr. Bottentuit.

Clinical Lectures on Albuminuria. By Thomas Grainger Stewart, M.D., Edin.

Clinical Atlas of Venereal and Skin Diseases. By Robert W. Taylor. Parts III and IV.

The Vest Pocket Anatomist. By C. Henri Leonard, M.D.

Transactions of the Association of American Physicians. Vol. III.

Pathology and Treatment of Displacements of the Uterus. By Dr. B. S. Schultze.

Guy's Hospital Reports. Vol. XLV. Edited by N. Davies-Colley, M.A., M.C., and W. Hale White, M.D.

Handbook of Materia Medica, Pharmacy, and Therapeutics. By Cuthbert Bowen, M.D., B.A.

Wood's Medical and Surgical Monographs. Volume I, Number 1: The Pedigree of Disease. By Jonathan Hutchinson, F.R.S.; Common Diseases of the Skin. By Robert M. Simon, M.D.; Varieties and Treatment of Bronchitis. By Dr. Ferrand. Number 2: Gonorrhœal Infection in Women. By William Japp Sinclair, M.A., M.D.; On Giddiness. By Thomas Grainger Stewart, M.D.; Albuminuria in Bright's Disease. By Dr. Pierre Jaenton.

### *PAMPHLETS RECEIVED.*

Diseases of the Nose and Pharynx and their Treatment. By W. Cheatham, M.D.

Malaria and the Causation of Periodic Fever. By Henry B. Baker, M.D.

The Medical Bulletin Visiting List for 1889. F. A. Davis, Philadelphia.

The Philosophy of Memory. By D. T. Smith, M.D.

The President's Annual Address. By Robert Battey, M.D.

On the Circular Polarization of Certain Tartrate Solutions. I. By J. H. Long.

New Form of Posterior Colporrhaphy. By J. H. Kellogg, M.D.

Experimental Researches respecting the Relation of Dress to Pelvic Diseases in Women. By J. H. Kellogg, M.D.

Report of Forty-eight Cases of Alexander's Operation. By J. H. Kellogg, M.D.

The Etiology of Dipsomania. By Lewis D. Mason, M. D.

The Radical Cure of Hernia. By Morris H. Henry, M.A., M.D.

Extremes in Altitude in Southern California. By Walter Lindley, M.D.

Transactions of the American Otological Society. 1888. Vol. 4. Part 2.

Annual Report of the Supervising Surgeon-General of the Marine-Hospital Service of the United States for the Fiscal Year 1888.

Pulmonary Consumption considered as a Neurosis. By Thomas J. Mays, M.D.

Two Cases of Gunshot Wound of the Abdomen. By N. Senn, M.D.

Inflation of the Stomach with Hydrogen Gas in the Diagnosis of Wounds and Perforations of this Organ. By N. Senn, M.D.

The Failure of Dr. J. B. Thomas's Treatment of Urethral Stricture by Electrolysis. By Robert Newman, M.D.

Treatment of Peritonitis by Abdominal Section. By L. S. McMurtry, M.D.

Electricity *vs.* Tait, or the Use of Electricity in Inflammation as Found in Gynæcology. By Geo. F. Hurlburt, M.D.

Case of Typhlitis, with Double Perforation of the Cæcum, and Peritonitis. By L. S. McMurtry, M.D.

Preliminary Report of an Operation for the Formation of an Artificial Pupil through the Sclerotic Coat of the Eyeball. By George Strawbridge, M.D.

The Influence which the Discovery of Cocaine has Exerted upon Ophthalmic Surgery. By Samuel Theobald, M.D.

Is Astigmatism a Factor in the Causation of Glaucoma? By Samuel Theobald, M.D.

The Histology and Surgical Treatment of Uterine Myoma. By Henry O. Marcy, M.D.

The Climate of the Southern Appalachians. By Henry O. Marcy, M.D.

Placental Development. By Henry O. Marcy. M.D.

The Case of the Emperor Frederick. Reports of the German Physicians and by Sir Morell Mackenzie.

The Anatomy and Pathology of the Thymus Gland. By A. Jacobi, M.D.

How Microörganisms Enter the Body. By George N. Kreider, M.D.

Cases in Orthopædic Surgery. By Ap Morgan Vance, M.D.

Femoral Osteotomy for Correction of Deformity from Hip-joint Disease. By Ap Morgan Vance, M.D.

Osteotomy for Anterior Curves of the Legs. By DeForest Willard, M.D.

COLLABORATORS:

CHICAGO:

Professor E. ANDREWS, M.D., LL.D.
Professor J. BARTLETT, M.D.
Professor W. T. BELFIELD, M.D.
Professor F. BILLINGS, M.D.
Professor W. H. BYFORD, A.M., M.D.
Professor L. CURTIS, A.M., M.D.
Professor N. S. DAVIS, Jr., A.M., M.D.
Professor O. C. DE WOLF, A.M., M.D.
Professor C. FENGER, M.D.
Professor J. N. HYDE, A.M., M.D.
Professor E. F. INGALS, A.M., M.D.
Professor F. S. JOHNSON, A.M., M.D.
Professor H. A. JOHNSON, M.D., LL.D.
Professor C. W. PURDY, M.D.
Professor C. G. SMITH, A.M., M.D.
Professor E. WING, A.M., M.D.
Professor E. C. DUDLEY, A.B., M.D.

MILWAUKEE, WIS.:
Professor N. SENN, M.D., Ph.D.

WASHINGTON, D. C.:
S. O. RICHEY, M. D.

UNITED STATES ARMY:
Surgeon D. L. HUNTINGTON, M.D.

UNITED STATES NAVY:
Surgeon-General J. M. BROWNE, M.D. Medical-Director A. L. GIHON, A.M., M.D.

MARINE HOSPITAL SERVICE:
Surgeon S. T. ARMSTRONG, M.D., Ph.D.

